# Insights into the Enhancement of the Poly(ethylene terephthalate) Degradation by FAST-PETase from Computational Modeling

**DOI:** 10.1021/jacs.3c04427

**Published:** 2023-08-16

**Authors:** Rafael García-Meseguer, Enrique Ortí, Iñaki Tuñón, J. Javier Ruiz-Pernía, Juan Aragó

**Affiliations:** † Instituto de Ciencia Molecular (ICMol), Universitat de València, Catedrático José Beltrán 2, 46980 Paterna, Spain; ‡ Departamento de Química Física, Universitat de València, 46100 Burjassot, Spain

## Abstract

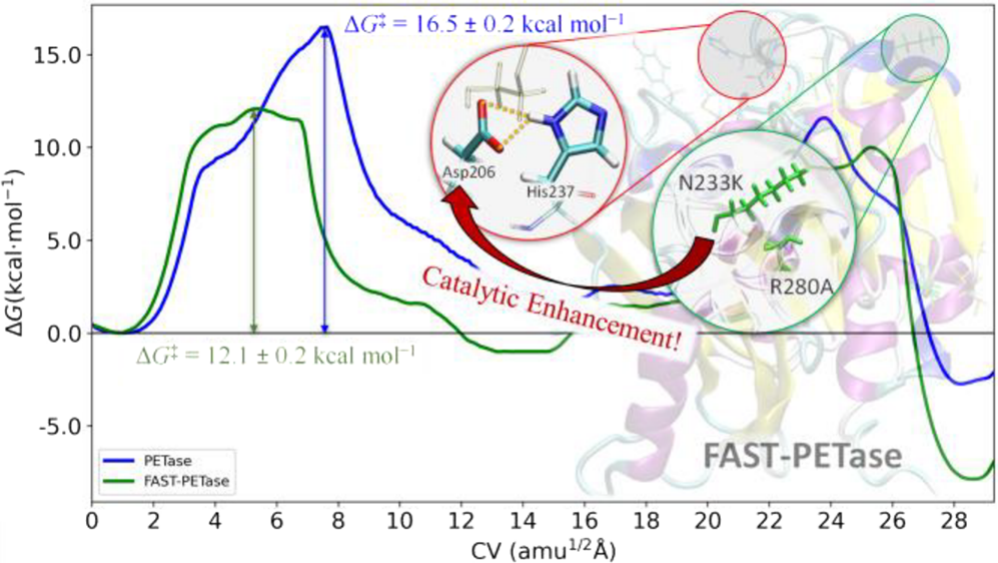

Polyethylene terephthalate (PET) is the most abundant polyester plastic, widely used in textiles and packaging, but, unfortunately, it is also one of the most discarded plastics after one use. In the last years, the enzymatic biodegradation of PET has sparked great interest owing to the discovery and subsequent mutation of PETase-like enzymes, able to depolymerize PET. FAST-PETase is one of the best enzymes hitherto proposed to efficiently degrade PET, although the origin of its efficiency is not completely clear. To understand the molecular origin of its enhanced catalytic activity, we have carried out a thorough computational study of PET degradation by the FAST-PETase action by employing classical and hybrid (QM/MM) molecular dynamics (MD) simulations. Our findings show that the rate-limiting reaction step for FAST-PETase corresponds to the acylation stage with an estimated free energy barrier of 12.1 kcal mol^–1^, which is significantly smaller than that calculated for PETase (16.5 kcal mol^–1^) and, therefore, supports the enhanced catalytic activity of FAST-PETase. The origin of this enhancement is mainly attributed to the N233K mutation, which, although sited relatively far from the active site, induces a chain folding where the Asp206 of the catalytic triad is located, impeding that this residue sets effective H-bonds with its neighboring residues. This effect makes Asp206 hold a more basic character compared to the wild-type PETase and boosts the interaction with the protonated His237 of the catalytic triad in the transition state of acylation, with the consequent decrease of the catalytic barrier and acceleration of the PET degradation reaction.

## Introduction

Plastics are ubiquitous materials in our daily lives due to their excellent durability, lightness, and low cost. The total production of plastics reached 390.7 million tons in 2021.^1^ However, only 14% of produced plastics is recycled, and the rest is incinerated (14%), deposited in a landfill (40%), or leaked to the environment (32%).^2^ Considering that plastics have a high chemical and biological stability, they can remain in the environment for hundreds of years,^3^ becoming a threat to terrestrial and marine ecosystems. Moreover, in recent studies, the presence of microplastics in seafood and foodstuff has been clearly documented, exposing humans to contaminated food with an impact on human health that is not fully understood yet.^4^ For all this, the development of new technologies that allow us to reduce, reuse, and recycle our plastic residues efficiently is a worldwide challenge for our current society.

**Figure 1:**
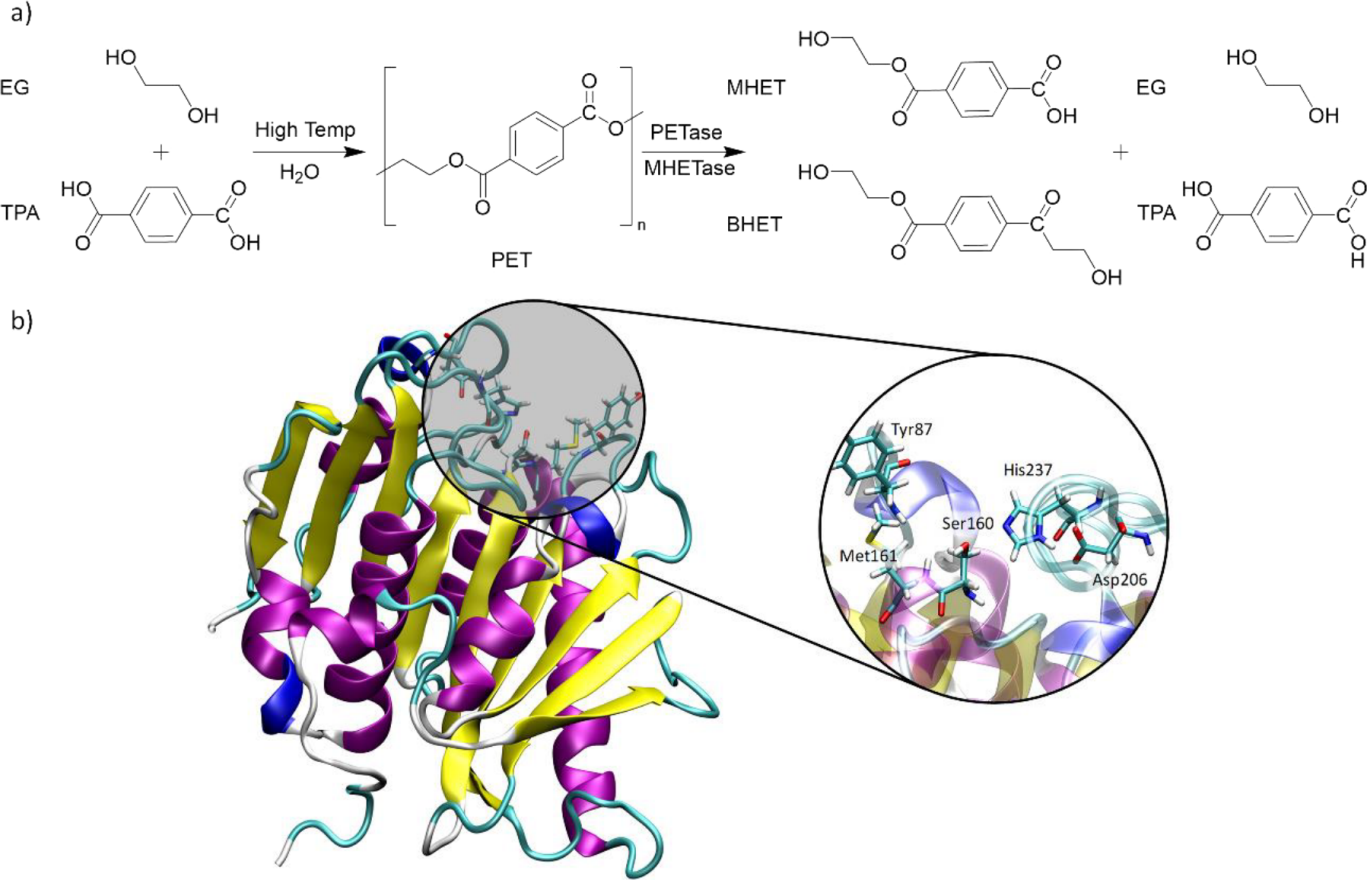
(a) Schematic representation of the polymerization of PET by its constituting monomers TPA and EG and its degradation by PETase and MHETase to the MHET, BHET, TPA, and EG products. (b) Structure of PETase (PDB ID: 6EQE). The enzyme exhibits nine β-strands (yellow) and seven α-helices (purple) in line with other hydrolases. A magnification of the active site highlighting the most important amino acids involved in the PET-degrading mechanism.

Polyethylene terephthalate (PET), obtained from the polycondensation of terephthalic acid (TPA) and ethylene glycol (EG), is likely to be the most common thermoplastic polymer whose ester group confers superior resistance to (bio)degradation (Figure 1a). PET is widely used in the food industry as liquid and food containers but also as clothing fibers.^5^ Unfortunately, it is also one of the most discarded plastics after one use (e.g., plastic bottles for water).^6^ Currently, the recycling of PET is partially achieved by employing a mechanical transformation from which clothing fibers can be obtained and reused for the textile industry. Nevertheless, the complete degradation or depolymerization of PET to its original components (TPA and EG) by chemical methods (chemical recycling) is an expensive (both economically and energetically) industrial process compared to its production from fossil fuel monomers.^7,8^ Faced with this problem, a more ecological alternative is the enzymatic biodegradation of PET.^9,10^ This biotechnological recycling route uses biocatalysts (i.e., enzymes) to hydrolyze the ester-type bonds present in PET and thus recover the starting TPA and EG monomers for further re-polymerization. This green technology is, therefore, very attractive since it allows us not only to reach the desired closed-loop recycling of PET (circular economy) but also to meet the Sustainable Development Goals. Although the initial degradation yields for PET-hydrolyzing enzymes were low for industrial implementation,^9,11−13^ new scientific advances in recent years foresee a promising future for enzymatic biorecycling of PET and other plastics.^10,14−21^

**Scheme 1:**
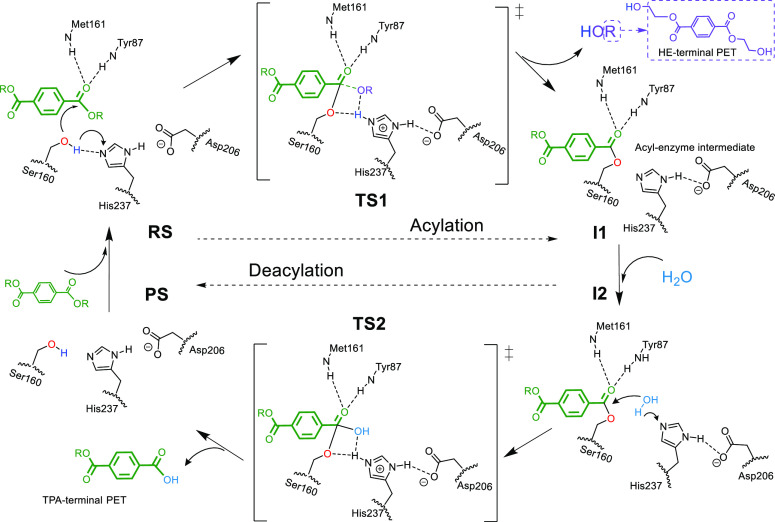
Schematic Representation of the PETase’s Reaction Mechanism

An important breakthrough was achieved by Yoshida and collaborators with the discovery of a bacterium (*Ideonella Sakaiensis* 201-F6) able to degrade and assimilate PET as its sole source of carbon and energy.^14^ Two enzymes (PETase and MHETase) were identified to be responsible for the depolymerization of PET into its original building blocks, TPA and EG (Figure 1a). Among the two enzymes, PETase is the enzyme that does the hardest work by recurrently decomposing the PET polymeric chain into the mono (2-hydroxyethyl) terephthalate (MHET), with trace amounts of TPA and bis(2-hydroxyethyl) terephthalate (BHET) as secondary products. Closing the catalytic process, MHETase then hydrolyzes MHET into TPA and EG monomers. In particular, PETase from *Ideonella Sakaiensis* is an interesting target due to its capability of degrading PET at moderate temperatures (30 °C) more efficiently than other PET-hydrolyzing enzymes,^14^ and has received considerable attention in the scientific community. Initially, scientific efforts were focused on the characterization of the PETase structure and its underlying PET-degrading mechanism. Almost in parallel, Han et al.^22^ and Joo et al.^23^ were able to identify the active site of PETase, in which the PET substrate was accommodated, as well as the relevant amino acids responsible for the enzyme:PET complex and the biocatalytic process (Figure 1b). Both studies revealed that the active site of PETase holds a typical catalytic triad (Ser160-His237-Asp206), in line with other hydrolases,^24^ two amino acids (Met161 and Tyr87), able to stabilize the oxygen of the PET carbonyl group by H-bonds (oxyanion hole), and an aromatic amino acid (Trp185), which can interact by π-stacking with a benzene ring of PET (Figure 1b). Austin et al. further confirmed the initial structural analysis after crystalizing the structure of the PETase enzyme at a more accurate resolution (0.92 Å).^25^ From this structural information, it was proposed that PETase follows the typical catalytic mechanism for cutinases, which comprises two steps (Scheme 1): an acylation process, with the formation of an intermediate acyl-enzyme complex and the release of a hydroxyethyl(HE)-terminal PET fragment, and deacylation, with the enzyme returning to its initial state and the formation of a TPA-terminal PET chain. This biocatalytic mechanism has been investigated at the molecular level by using quantum mechanics/molecular mechanics (QM/MM) simulations.^26−29^ First, Boneta et al.,^26^ by using semiempirical AM1/MM umbrella-sampling calculations with further corrections from density functional theory (DFT) with the M06-2X functional, described the mechanism as a two-step process in both acylation and deacylation via a tetrahedral enzyme-substrate intermediate and two transition states for each stage. In contrast, Jerves et al.,^27^ using a larger QM region described at the PBE level, predicted a single-step catalytic mechanism for both stages (acylation and deacylation) through a tetrahedral transition state. Although it is not relevant to this work, scientific advances concerning the elucidation of the structure and catalytic mechanism of MHETase have also been recently achieved.^30−32^

Apart from the mechanistic aspects of the PET biodegradation, the scientific spotlight has also been put on PETase engineering to obtain novel variants capable of working more efficiently and at a higher range of temperatures. Note that the PET biodegradation rate drops with the rising degree of crystallinity and, thus, the quest for thermostable PETase scaffolds able to maintain their PET-degrading activity at temperatures higher than 60 °C—temperature in which the glass transition of PET from a (semi)crystalline to amorphous phase occurs—is highly attractive for large-scale industrial applications. Likewise, efficient PETase scaffolds functioning at mild conditions, even with a pretreatment of PET, are also highly interesting for industrial recycling processes. With this in mind, several efficient PETase and cutinase-like variants have already been proposed in the last few years.^16,17,33,34^ Regarding the PETase mutants, Son et al. proposed, by using the structural information of the wild-type PETase, a rational protein engineering strategy to increase the thermostability and the PET degradation activity of the enzyme.^33^ In particular, they obtained a PETase scaffold (known as ThermoPETase), with only three mutations with respect to the wild-type homolog (S121E/D186H/R280A), in which the thermal stability was enhanced by ca. 9 °C and the PET degradation activity improved by 14-fold at 40 °C. From a different approach, Cui et al.^34^ computationally redesigned PETase to provide a new variant (DuraPETase) which, after its synthesis, exhibited a high melting temperature (a *T*_m_ value of 77 °C compared to that reported for PETase, *T*_m_ = 46 °C) and strikingly enhanced degradation toward semicrystalline PET films at mild temperatures (over 300-fold). Despite these interesting PETase mutants, the recent discovery of the variant proposed by Lu et al. (known as FAST-PETase) has meant an important breakthrough.^16^ The FAST-PETase enzyme, initially designed by machine learning techniques, contains only five mutations with respect to wild-type PETase and exhibits a superior catalytic activity in the 30–50 °C range compared to wild-type PETase and all other so far proposed mutants. Interestingly, FAST-PETase is able to completely biodegrade untreated and postconsumer PET waste from different products in 1 week. More challenging, it depolymerizes PET in the amorphous domains of untreated commercial water bottles and, under thermal pretreatment, an entire water bottle at 50 °C. This promising study opens the door for a closed-loop PET recycling process at the industrial scale, where the FAST-PETase enzyme is used to depolymerize PET and the recovered monomers are employed to resynthesize PET again.

Despite FAST-PETase’s success, the origin of its enhanced catalytic activity is not completely understood at the molecular level. Note that the five mutations in FAST-PETase, all of them out of the active site, seem to enhance the stability of the enzyme by extending the H-bonding network and establishing a favorable electrostatic salt bridge interaction. Therefore, it is not clear how these mutations can stabilize the initial enzyme:PET complex or the intermediate structures (transition states) that determine the catalytic mechanism and the reaction rate for PET biodegradation. That mechanistic information at the molecular level can be very helpful for the design of novel and enhanced FAST-PETase mutants but also for other PET-hydrolyzing enzymes. Herein, we seek to shed light on this knowledge gap from a computational perspective. Classical molecular dynamics (MD) simulations for the mutant FAST-PETase and the wild-type PETase enzymes and their respective enzyme:PET complexes were first performed to gain insight into the structural changes induced by the mutations in the FAST-PETase scaffold that might be important for the PET depolymerization. In a second step, multiscale QM/MM simulations were employed to characterize the catalytic mechanism of FAST-PETase in comparison with wild-type PETase to unveil the enhancement of the FAST-PETase activity. The free energy profiles (FEPs) calculated for the PET degradation (acylation and deacylation processes) allowed us to connect all intermediates and transition states from reactants to products.

## Computational Methods

### Building of (FAST-)PETase:PET Systems

**Scheme 2:**
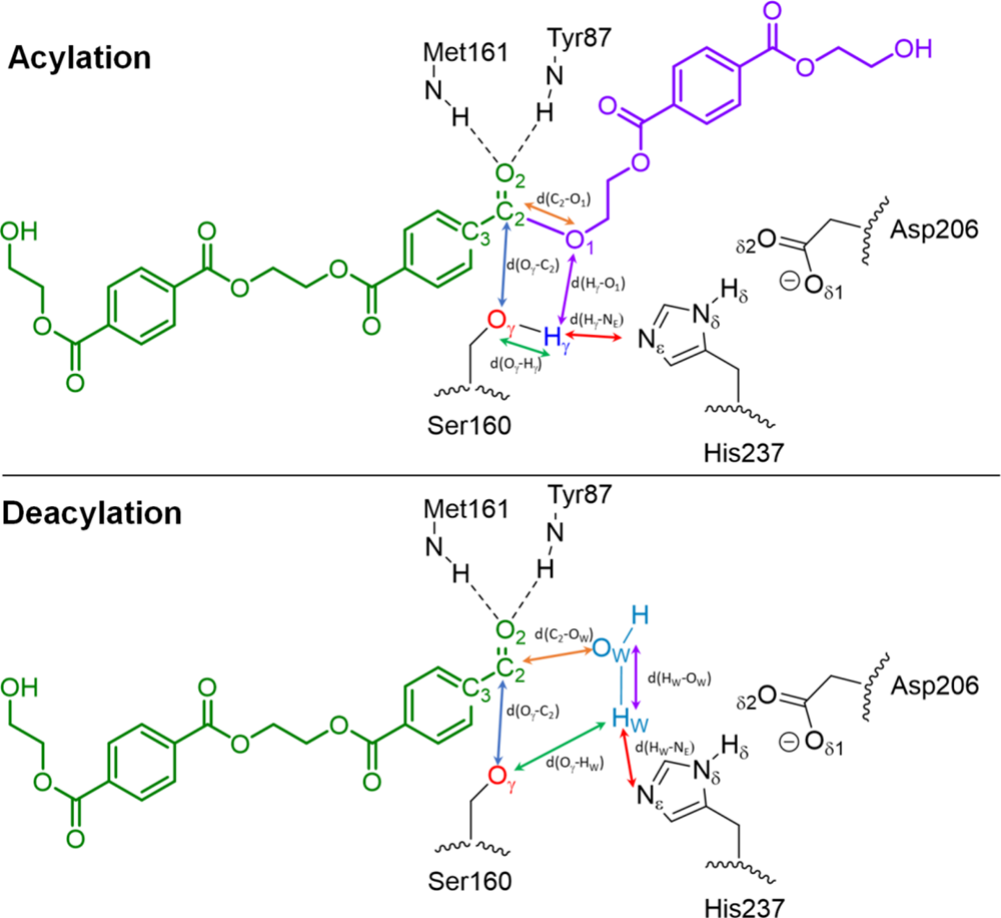
Subsystem Described at the Quantum-Mechanical Level (QM Part) The PET substrate is highlighted in green and purple. The relevant distances used to define the CV of the string method are depicted for both the acylation (top) and deacylation (bottom) steps of the mechanism.

The initial coordinates of the PETase enzyme were taken from the highest-resolved X-ray crystal structure (0.92 Å) of the apoenzyme available in the Protein Data Bank, with code 6EQE.^25^ For the FAST-PETase mutant, the structure available with code 7SH6 was selected.^16^ To calculate the adequate protonation states of titratable residues at pH 7.0, the PROPKA3.0 software tool was employed.^35,36^ We introduced the PET substrate model (a trimer) into the enzyme systems using the alignment with a PETase-substrate crystal structure (5XH3),^22^ in which the substrate corresponds to the 1-(2-hydroxyethyl) 4-methyl terephthalate. The PET trimer model used here is 2-hydroxyethyl-(mono-hydroxyethyl terephthalate)_3_, abbreviated as 2-HE(MHET)_3_ (Scheme 2). The PET model was described by the GAFF37 parameters using the Antechamber module of the AMBER20 software package^37^ and employing the RESP atomic point charges calculated at the HF/6-31G(d) level with the Gaussian 09 program.^38^ Once the enzyme:PET systems were built, the PET substrate was minimized by 2000 steps of the steepest descent method followed by the conjugate gradient method until the root mean square of the gradient was below 50 kcal mol^–1^ Å^–1^ in order to accommodate the substrate into the active site of both enzymes (FAST-PETase and PETase). Afterward, both systems were prepared using the *tleap* tool implemented in the AMBER20 software.^37^ The enzyme scaffolds (both the FAST-PETase mutant and wild-type PETase), described with the ff14SB force field implemented in AMBER20,^37^ were solvated into a box of TIP3P^39^ water molecules, with a buffer region of at least 12 Å from any protein substrate atom to the limits of the simulation box. We added six Cl^–^ anions to neutralize the total charge of the systems. Then, the resulting FAST-PETase:PET (PETase:PET) system was composed of 37,243 (37,247) total atoms: 3795 (3799) protein atoms, 76 PET atoms, 6 counterions, and 11,122 water molecules.

For both enzyme:PET systems, the protocol was the same. The entire system was minimized using 5000 steps of the steepest descent method followed by the conjugate gradient method. Initially, only the water molecules and counterpoise ions were minimized, keeping the rest of the system fixed. Then, the entire system, except for the protein’s backbone, was minimized. Finally, the entire system without restrictions was fully minimized until the root mean square of the gradient was below 10 kcal mol^–1^ Å^–1^. The system was later heated from 0 to 300 K using a heating rate of 1.7 K·ps^–1^. In order to equilibrate the system, 100 ns of classical MD simulations at 300 K with periodic boundary conditions were performed. The time step of the classical simulation was 1 fs using the SHAKE algorithm.^40^ The Particle Mesh Ewald method was employed to describe the long-range electrostatic interactions^41,42^ with a cutoff of 15 Å. We used the Berendsen barostat^43^ and the Langevin thermostat^44^ to control the simulation’s pressure and temperature, respectively. Free protein versions of FAST-PETase and PETase (i.e., without the PET substrate) were generated from the respective X-ray structures following the same computational procedure.

### Classical MD Simulations

To analyze the structural properties of the enzymes (FAST-PETase and PETase with and without the PET model), we run 1 μs of classical MD simulations with the same characteristics as the relaxation described in the previous section but with a time step of 2 fs (also using SHAKE) and saving points every 100 ps. By using the CPPTRAJ program,^45^ included in AMBER20, the root-mean-square deviation (RMSD) and the root-mean-square fluctuation (RMSF) for each protein were calculated.

From the simulations over the (FAST)-PETase:PET complexes, the binding energies of the PET model with the FAST-PETase and PETase enzymes were estimated by using the GBSA implicit solvent model^46^ using 1000 frames spaced by 1 ns from the trajectory with the MM-PBSA.py tool^47^ included in the AMBER20 program. The same program used to estimate the enzyme-substrate binding energy (MM-PBSA.py) also allowed us to split the binding energy into its fundamental contributions (internal and solvation).

The electrostatic potential felt by a selected atom was obtained by calculating the total energy of the whole system and subtracting the total energy of the system but with the charge of the selected atom set to zero, and the difference divided by its original charge.

### QM/MM MD Simulations

Finally, a QM/MM molecular dynamic simulation of 100 ps for the stable structures of the reaction mechanism (i.e., **RS**, **I1**, **I2**, and **PS**, Scheme 1) was performed. For acylation, the QM region was composed of a total of 129 atoms: (i) the full PET trimer, (ii) the catalytic triad (His237, Ser160, and Asp206), and (iii) the Tyr87 and Met161 residues (oxyanion hole) (Scheme 2, top). The semiempirical DFTB3 Hamiltonian^48^ implemented in AMBER20 was used for the QM part. For deacylation, the PET trimer was substituted by the dimer bonded to Ser160’s oxygen, and a water molecule (WAT) was introduced in the QM region to react with the acyl-enzyme complex in the active site (Scheme 2, bottom).

In order to explore the free energy landscapes associated with the chemical reaction, our implementation of the adaptive string method (ASM) was employed.^49^ In this method, *N* replicas of the system (the nodes of the string) evolve according to the averaged forces and stay equidistant, converging in such a way to the minimum free energy path (MFEP) in the space of arbitrary dimensionality. Once the string has converged, we define a single path using a path Collective Variable (path-CV) that measures the advance of the system along the MFEP. This path-CV represents the reaction coordinate used to trace the FEPs along the chemical reaction. We explored the MFEPs on a free energy hypersurface defined by a set of CVs formed by those variables (distances, angles, or torsions), showing relevant changes during the process under study. QM/MM MD simulations to define the beginning/end of our initial/final string guesses were performed. In line with the reaction mechanism, we divided the process into two strings: acylation and deacylation. Scheme 2 shows the relevant distances used to define the CV for each reaction stage. Note that we also included point-plane distances (to the plane formed by C_2_, O_1_, O_2_, and C_3_ atoms for acylation and C_2_, O_2_, C_3_, and O_γ_ for deacylation) in the CV to facilitate the string convergence. These distances were removed from the analysis, for clarity purposes, as they do not provide relevant mechanistic information.

Once the MFEP was converged, the CV reaction coordinate was defined and MD simulations (at least for 100 ps) were run at each node on the string using an umbrella potential automatically defined. Then, with the aid of the weighted histogram analysis method (WHAM),^50^ the potential of mean force (PMF) profiles were calculated.

We anticipate that the free energy barriers calculated with the current approximation for both acylation and deacylation in wild PETase are in good accord with other theoretical studies (Boneta et al.^26^ and Jerves et al.,^27^ see below). This indicates that the selected computational approximation is trustworthy for further studies of other PETase-like mutants. Nevertheless, the comparison between the different QM/MM MD schemes can highlight some differences interesting for future theoretical treatments of novel PETase-like variants. Jerves et al.^27^ accurately studied the PET degradation by the wild-type PETase by means of a robust QM/MM umbrella-sampling method with a large QM region at the PBE level including 146 atoms (a PET dimer; the catalytic tSer131-Hist208-Asp177 triad; the Tyr58 and Met132 residues responsible for the oxyanion hole; Trp156 to set π–π interactions with the PET fragment; and the extra residues Ser178, Ile179 and Ala180). This study predicted a single-step catalytic mechanism for both stages (acylation and deacylation) through a tetrahedral transition state consistent with the experimental mechanism initially proposed by Han et al.^22^ and Joo et al.^23^ Our approximation, with a reduced QM region (a PET trimer, the catalytic triad Ser160-His237-Asp206 and the Tyr87 and Met161 residues) and the ASM, also predicts a single-step catalytic mechanism in line with the most accurate theoretical and experimental studies. In contrast, Boneta et al.^26^ obtained a two-step mechanism for both acylation and deacylation, which seems not to be totally in accord with the previous studies. They employed semiempirical AM1/MM umbrella-sampling calculations with further corrections from DFT (M06-2X functional) for a small QM region (a PET dimer and the catalytic triad). This comparison reveals that the medium-size QM region selected here (treated at the DFTB3 level) in combination with the ASM for the free energy landscapes is a good approach with a reasonable balance between precision and computational cost.

## Results and Discussion

### Structural Analysis and Binding Energies from Classical Simulations

**Figure 2:**
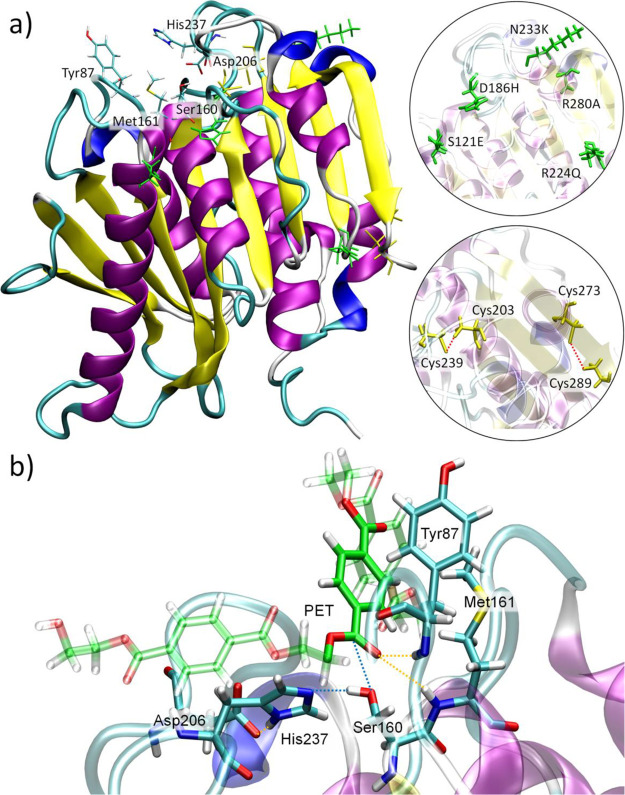
(a) Structure of FAST-PETase where the secondary structure’s features are colored for β-strands (yellow) and α-helices (purple). The active site is depicted with the most relevant residues (labeled as sticks colored by element). Mutations and disulfide bonds are colored in green and yellow sticks, respectively, and labeled in the right close-ups. (b) Close-up view of the FAST-PETase active site with the PET substrate.

The crystal structure of the FAST-PETase enzyme reveals that the structure of FAST-PETase is quite similar to that found for the wild-type PETase variant, being organized in a set of nine β-strands and seven α-helices (Figure 2). Similar to PETase, the active site of FAST-PETase is located on the enzyme surface (in a surface cleft) and holds the canonical catalytic Ser-His-Asp triad (hereafter, Ser160-His237-Asp206), which is essential for the catalytic activity.^24^ FAST-PETase also maintains the two characteristic disulfide bonds (Cys203-Cys239 and Cys289-Cys273) of PETase, which endows the enzyme with additional stability. Although the disulfide bond Cys203-Cys239 is located near the active site, it is still flexible enough to effectively accommodate the rigid PET substrate.

As mentioned above, the FAST-PETase variant only contains five mutations with respect to the wild-type PETase (S121E/D186H/R224Q/N233K/R280A). In the crystal structure, the first two mutations (the replacement of Ser121 and Asp186 by Glu and His, respectively) are located relatively close to the active site (ca. 7 Å), enabling to establish a more extended H-bond network between Glu121, H186, and Asn172 through two water molecules.^16^ The substitution of Arg224 by Gln did not cause any impact in terms of H-bonds; i.e., the H-bond between Arg224 and Ser193 for PETase is lost, and a new bond is formed between the shorter Gln224 peptide and Ser192 in FAST-PETase. The N233K mutation, which substitutes the neutral Asn233 by a positively charged Lys, gives rise to an intramolecular salt bridge with the close Glu204 by an optimal electrostatic interaction. Note that N233K is sited relatively near the His237 and Asp206 of the catalytic triad (ca. 11 Å) although it is still far from the active site. Interestingly, N233K is likely to be the most important mutation for the PET-degrading reaction since that mutation has been incorporated in several PET-degrading enzymes (e.g., LCC, ICCM, and Cut190) and, in all of these mutants, the hydrolytic activity has improved compared to their respective previous scaffolds.^16^ Finally, the R280A mutation, where Arg280 is replaced by Ala, is found to be close to the N233K mutation (ca. 5 Å) and at ca. 15 Å of the active site. That mutation is present not only in FAST-PETase but also in the ThermoPETase variant,^33^ and its role was ascribed to a better binding with the PET substrate.

All the mutations described above are found to be relatively distant from the active site (>7 Å) and, in principle, their direct impact on the biocatalytic mechanism is not evident. To gain insight into the structural changes induced by the mutations that can be relevant to the reaction, classical MD simulations of 1 μs were performed for the free enzymes and their respective enzyme:PET complexes. In particular, the changes caused by the mutations in their local environments (intermolecular contacts) were analyzed along the dynamics and, to get a broader perspective, the mobility of each protein residue (RMSF analysis) was also examined (see below).

As mentioned above, mutations D186H and S121E seem to promote an extended H-bonding network between Glu121, H186, and Asn172 through two bridging water molecules. Note that the Glu121, H186, and Asn172 residues are sited on the enzyme surface and, thereby, are easily accessible to the water solvent. Our classical MD simulations reveal that this H-bonding network is highly dynamic, with a steady formation/breaking of the intermolecular H-bonds between the respective peptides and close water molecules. From our analysis of the H-bonds over the classical trajectories, we can observe that the solvent-mediated-bridge between Glu121 and His186 is maintained and slightly increased (the fraction of frames forming the bridge is 0.12 compared to the bridge formed by Ser121 and Asp186 in the wild-type variant which is 0.10). Likewise, the solvent-mediated-bridge between Glu121 and Asn172 is strengthened in FAST-PETase (fraction of 0.10) compared to PETase, where there is hardly any solvent-mediated-bridge between Ser121 and Asn172 (fraction of 0.002). Interestingly, the effective interaction between Glu121, H186, and Asn172 via water-mediated H-bonds strengthens the interaction between two β-strands and an α-helix. Our simulations, in line with the X-ray structure, therefore support that the D186H and S121E mutations may be responsible for the enhanced thermal stability due to the more extended H-bonding network.

The previously discussed mutations (D186H and S121E) together with R280A are indeed the mutations incorporated in the wild-type enzyme to give rise to the ThermoPETase variant.^33^ The new and small-sized Ala280 forms an effective H-bond within the β-strand with Asn277 with an averaged distance of 2.3 ± 0.4 Å. This H-bond is not observed between Arg280 and Asn277 for the wild-type PETase (6.1 ± 0.2 Å) because Arg280 is interacting with the vicinal residues of the contiguous α-helix. As briefly mentioned above, the enhanced PET-degrading activity caused by the R280A mutation was associated with an extension of a subsite (subsite IIc) to accommodate the PET polymer more easily.^33^ That was explained because the incorporation of the short Ala280 residue generates a hydrophobic and nonprotruding cleft. Unfortunately, we cannot evaluate the enhancement of the enzyme-PET interaction due to this mutation because our PET model (a trimer) is not large enough to reach the subsite where the Ala280 residue is placed.

For the R224Q mutation, our simulations, in good accord with the crystal structure analysis, display that Gln224 is able to establish a weak H-bond (2.9 ± 0.2 Å) with Ser192 along the trajectory. Gln224 seems not to promote the formation of extra H-bonds with other neighboring residues. It is therefore difficult to unveil the role of this mutation from the computational results since, on the one hand, the mutation is far from the active site (ca. 22 Å) to have a direct influence on the PET-degrading reaction and, on the other hand, the lack of extra H-bonds with vicinal residues would not improve the thermostability of the enzyme. This conclusion is however in line with the experimental outcomes since all the PETase-like variants (wild-type, ThermoPETase, and DuraPETase) with only this mutation hardly improve the PET degradation, and thus, this mutation seems to be less relevant.

We now turn our attention to the N233K mutation, which is by far the key mutation for the enhanced PET degradation ability not only in FAST-PETase but also in other related variants.^16^ Previously, Lu et al. claimed that the incorporation of Lys233 (positively charged) upon the N233K mutation favored an attractive electrostatic interaction (salt bridge) with the Glu204 residue (negatively charged). Our simulations clearly indicate an opposite behavior; i.e., the salt bridge between Lys233 and Glu204 is not preserved along the dynamics. Lys233 and Glu204 are significantly separated with an average distance between the two oxygen atoms of Glu204 and the positively charged nitrogen of Lys233 of 11.0 ± 3.0 Å (Figure S1). We have also analyzed the potential H-bonds between the hydrogens attached to the positively charged nitrogen of Lys233 with any of the two oxygen atoms of Glu204 for FAST-PETase, and only a small fraction of frames with H-bonds (6%) is predicted, which contrasts to the fraction (34%) calculated for the wild-type enzyme between Asn233 and Glu204. What actually occurs along the dynamics is that Lys233, which is placed on the enzyme surface, is highly stabilized by effectively interacting with the accessible water molecules of the solvent.

**Figure 3:**
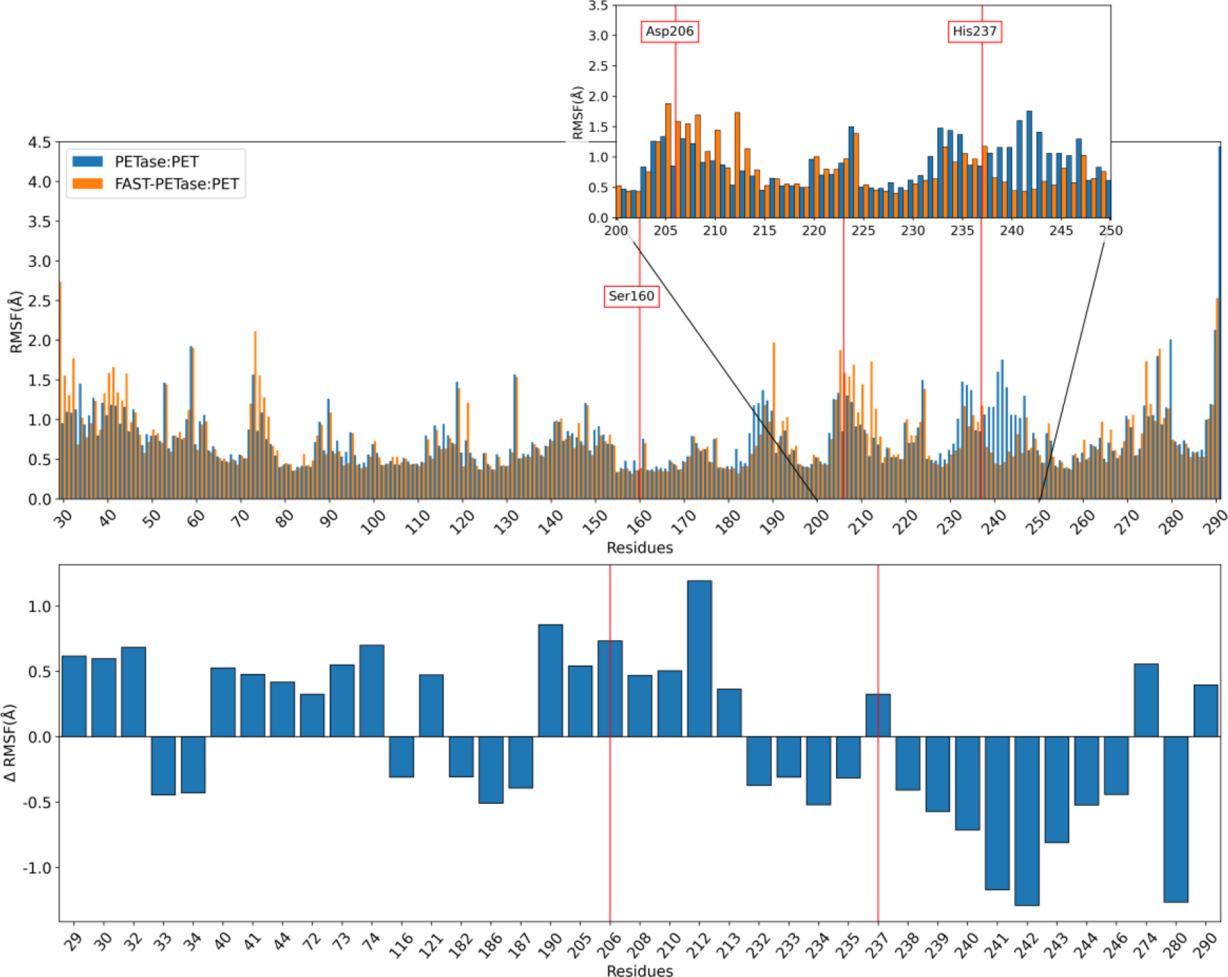
Plot of the RMSF calculated for each residue of PETase:PET and FAST-PETase:PET complexes (top) and its difference ΔRMSF (ΔRMSF = RMSF_FAST-PETase:PET_–RMSF_PETase:PET_) (bottom). For the ΔRMSF plot, only the residues with differences greater than 0.3 Å in absolute value are shown along the abscissa axis. The catalytic triad residues are marked with red lines.

Since the previous structural analysis throughout the dynamics for the free enzymes did not provide conclusive insights concerning the enhanced catalytic activity of FAST-PETase, the dynamics of the enzyme:PET complexes were also analyzed. Interestingly, the N233K mutation causes two important effects that can be visualized by representing the RMSF of each residue for the FAST-PETase:PET and PETase:PET complexes and the difference (ΔRMSF) between both enzyme:PET complexes (Figure 3). The main effect is the decreased mobility of the residues that are directly linked to Lys233 (residues 232–246) except for His237, which is accompanied by the increased mobility of the residues bonded to Glu204, among which is Asp206. The increased mobility of Asp206 in the FAST-PETase:PET complex is associated with a change in the chain folding (residues 206–210, Figure S2), causing less effective H-bonds interactions of the Asp206 with its neighboring residues (Ile208 and Ala209) compared to the PETase:PET complex (Figure S3). We anticipate that the difficulty of Asp206 to set H-bonds with its vicinal residues in the FAST-PETase:PET complex makes Asp206 hold a more basic character supported by the higher electrostatic potential felt by the O_δ1_ and O_δ2_ atoms (141.0 ± 8.0 and 140.0 ± 8.0 kcal mol^–1^ |e|^–1^, respectively) compared to the PETase:PET complex (135.0 ± 7.0 and 129.0 ± 7.0 kcal mol^–1^ |e|^–1^, respectively, Figure S4). As explained below, the increased basicity of Asp206 in FAST-PETase:PET is crucial to strengthen the interaction with His237 and stabilize the transition state during the acylation stage (next section). Therefore, the mutation N233K, which is relatively far from the active site, has an effect on the residues of the catalytic triad, in particular over His237 and Asp206, after removing the interaction with Glu204.

For the enzyme:PET complexes, the binding energies between the PET model and the enzyme scaffolds were also computed from the classical simulations (see the Computational Details). The five mutations inserted in FAST-PETase seem to have a small impact on the enzyme-PET interaction. The predicted binding energies are similar for the mutant and the wild-type, the binding energy being slightly smaller for the FAST-PETase:PET complex (−28.0 ± 5.0 kcal mol^–1^) than that for PETase:PET (−32.0 ± 4.0 kcal mol^–1^). Nevertheless, a further analysis by decomposing the binding energy into two contributions (enzyme:substrate and solvation) reveals that the interaction between the PET fragment and the enzyme is stronger for FAST-PETase (−49.4 ± 10.1 kcal mol^–1^) than for PETase (−37.5 ± 4.4 kcal mol^–1^). The contribution from solvation indeed equilibrates the binding energies for both enzyme:PET systems with a more unfavorable solvation energy for the FAST-PETase:PET complex compared to PETase:PET (21.0 ± 5.0 and 5.0 ± 0.9 kcal mol^–1^, respectively). These findings suggest that the PET substrate is better accommodated inside the active site for FAST-PETase but less exposed to the solvent. These energetic results can be explained by the structural analysis. The insertion of PET in FAST-PETase is accompanied by a cleft narrowing of the active site (13.0 ± 0.4 Å) compared to the wild-type enzyme (14.5 ± 0.5 Å, Figure S5). Additionally, this shrinkage of the active cleft in FAST-PETase also reduces the number of water molecules accessible in the active site. This is supported by the radial distribution function of water molecules around Ser160 (Figure S6) and the number of water molecules within the cavity formed by the catalytic triad (Figure S7) for both FAST-PETase:PET and PETase:PET complexes. The smaller number of water molecules surrounding Ser160 and also within the cavity of the catalytic triad for FAST-PETase indicates a more favorable preorganized state compared to PETase, which would be favorable for the rate of the PET-degrading reaction as occurs in other biocatalysts. Desolvation of the Michaelis complex may contribute to a decrease in the activation free energy of the catalytic process.^51^ Finally, it should be noted that the reduction of accessible water molecules for FAST-PETase is not due to a significant change in the hydrophobicity of the active site as no mutation is present in the site (Figure S8).

### Biocatalytic Mechanism of the PET Degradation

**Figure 4:**
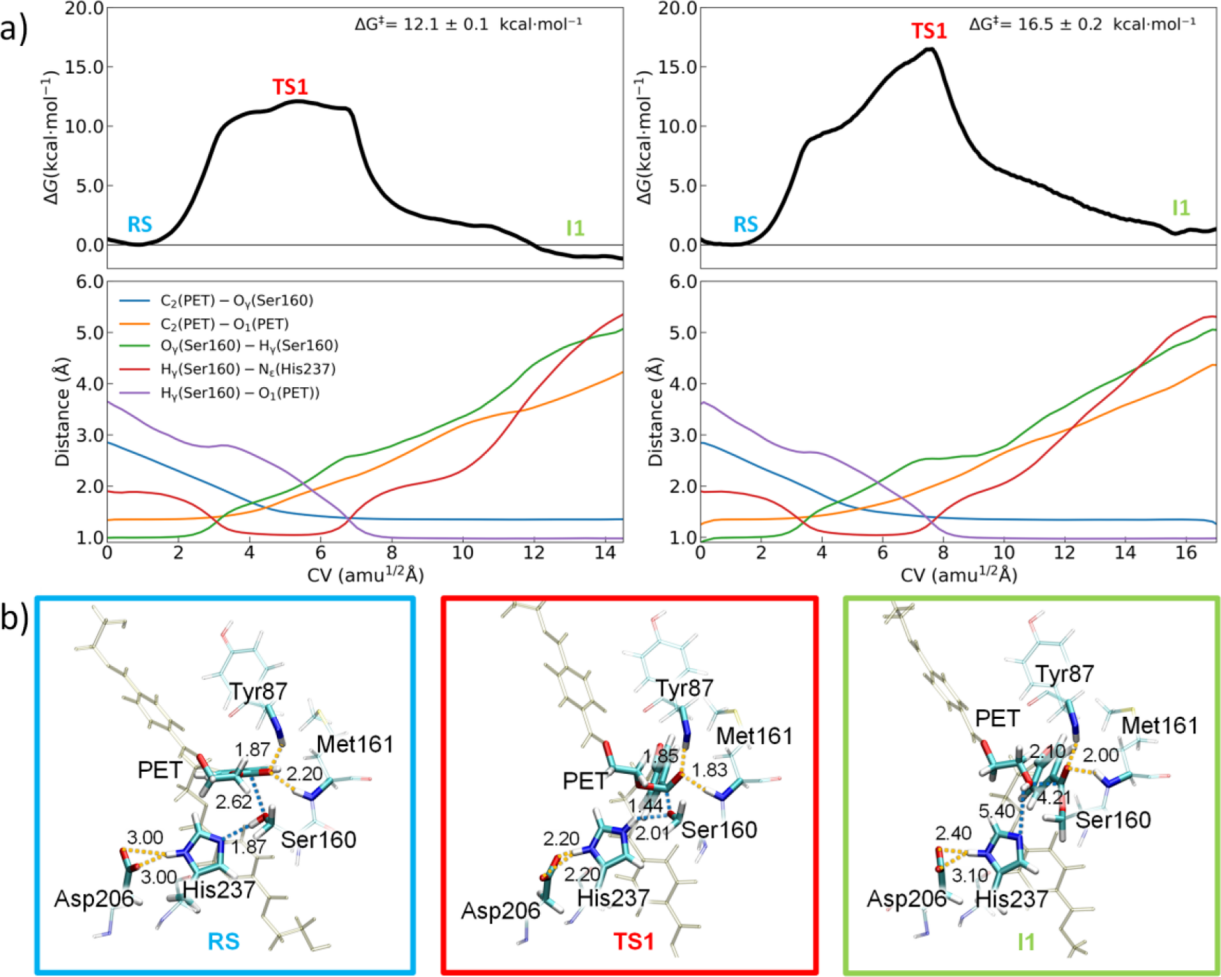
(a) (Top) Free energy profiles calculated along the CV for the acylation mechanism of FAST-PETase (left) and PETase (right). The values of the free energy barriers (Δ*G*^‡^) are given. (Bottom) Evolution of the most relevant variables used in the definition of the CV along the MFEP. (b) Representative structures (atom distances in Å) of the active site at the relevant points (**RS**, **TS1**, and **I1**) for FAST-PETase. Structures at the relevant points for PETase are similar and shown in Figure S9.

**Figure 5:**
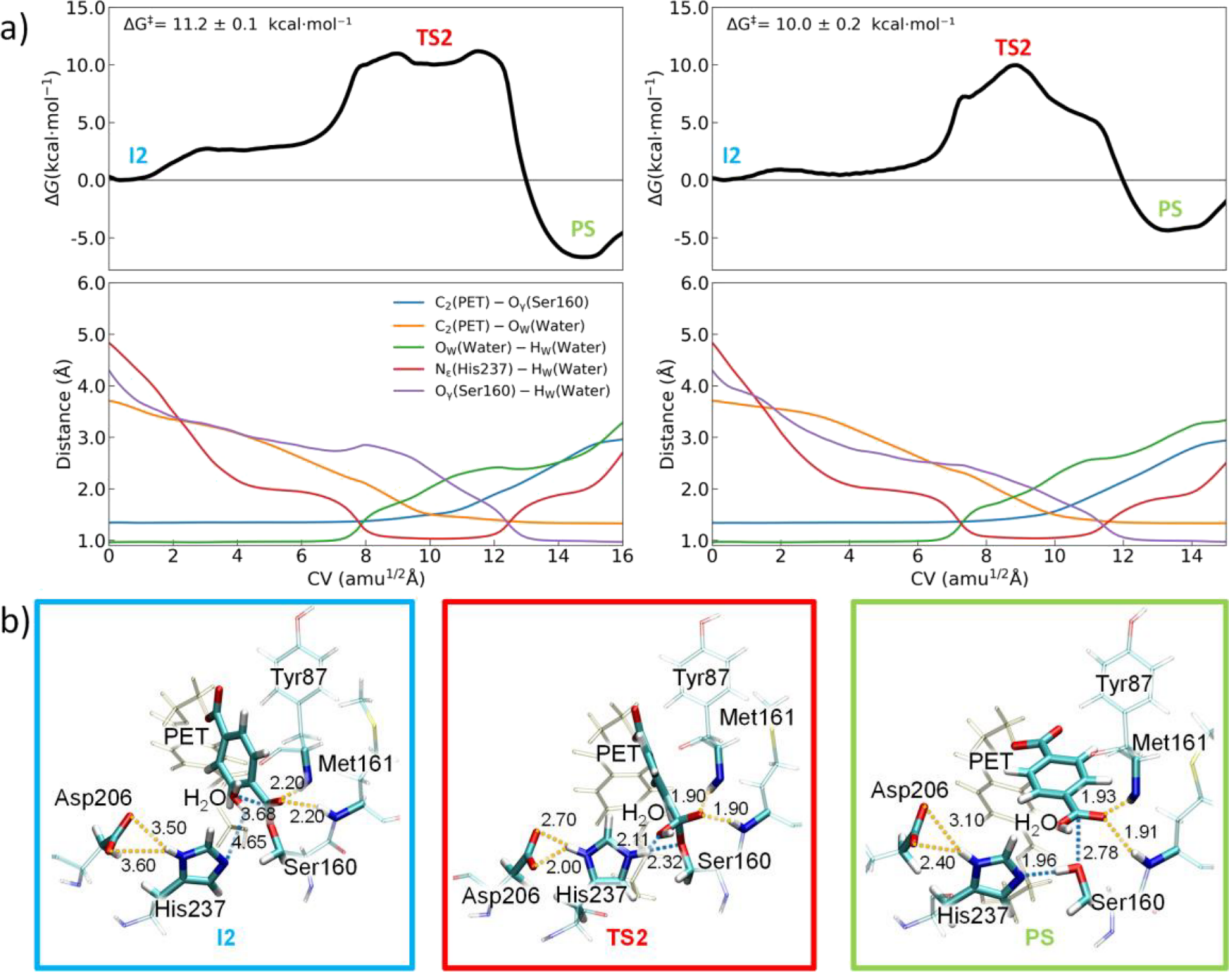
(a) (Top) Free energy profiles calculated along the CV for the deacylation stage of FAST-PETase (left) and PETase (right). The values of the free energy barriers (Δ*G*^‡^) are given. (Bottom) Evolution of the most relevant variables used in the definition of the CV along the MFEP. (b) Representative structures (atom distances in Å) of the active site at the relevant points (**I2**, **TS2**, and **PS**) for FAST-PETase. Structures at the relevant points for PETase are similar and shown in Figure S9.

Umbrella-sampling simulations along the path-CV defined by the ASM were performed to estimate the FEPs for the mutant FAST-PETase and the wild-type PETase enzymes for the acylation and deacylation stages (Figures 4 and 5, respectively). Figures 4 and 5 also display the evolution of the most important structural parameters (distances) involved in the path-CV along MFEPs, whereas Table 1 gathers the average values of those selected distances for the most relevant species along the reaction paths.

**Table 1:** Average Distances (Å) Calculated for the most Relevant Species (RS, TS1, I1, I2, TS2, and PS) along the Acylation-Deacylation Mechanism

acylation						
	FAST-PETase			PETase		
distances	RS	TS1	I1	RS	TS1	I1
C_2_(PET)–O_γ_(Ser160)	2.62 ± 0.06	1.44 ± 0.04	1.35 ± 0.03	2.67 ± 0.09	1.40 ± 0.04	1.35 ± 0.03
C_2_(PET)–O_1_(PET)	1.35 ± 0.03	1.86 ± 0.07	4.21 ± 0.08	1.35 ± 0.03	1.96 ± 0.06	4.6 ± 0.2
O_γ_(Ser160)–H_γ_(Ser160)	1.00 ± 0.03	2.01 ± 0.13	5.1 ± 0.2	0.99 ± 0.03	2.6 ± 0.2	5.3 ± 0.3
N_ε_(His237)–H_γ_(Ser160)	1.87 ± 0.09	1.04 ± 0.03	5.4 ± 0.2	1.92 ± 0.14	1.21 ± 0.11	6.8 ± 0.6
H_γ_(Ser160)–O_1_(PET)	3.3 ± 0.2	2.04 ± 0.14	0.97 ± 0.02	3.3 ± 0.2	1.34 ± 0.14	0.98 ± 0.03
O_δ1_(Asp206)–H_δ_(His237)	3.0 ± 1.2	2.2 ± 0.3	3.1 ± 0.6	2.2 ± 0.3	2.0 ± 0.2	2.8 ± 0.7
O_δ2_(Asp206)–H_δ_(His237)	3.0 ± 1.0	2.2 ± 0.3	2.4 ± 0.7	3.1 ± 0.3	2.8 ± 0.3	3.1 ± 0.6
H(Met161)–O_2_(PET)	2.2 ± 0.2	1.83 ± 0.10	2.0 ± 0.2	2.2 ± 0.2	1.89 ± 0.13	2.1 ± 0.2
H(Tyr87) −O_2_(PET)	1.87 ± 0.13	1.85 ± 0.11	2.1 ± 0.2	1.94 ± 0.14	1.83 ± 0.11	2.0 ± 0.2

deacylation						
	FAST-PETase			PETase		
distances	I2	TS2	PS	I2	TS2	PS
C_2_(PET) −O_γ_(Ser160)	1.34 ± 0.03	1.49 ± 0.05	2.78 ± 0.05	1.34 ± 0.03	1.44 ± 0.04	2.55 ± 0.04
C_2_(PET) −O_w_(water)	3.68 ± 0.05	1.49 ± 0.05	1.33 ± 0.03	3.67 ± 0.07	1.80 ± 0.05	1.34 ± 0.03
O_w_(Water)–H_w_(water)	0.97 ± 0.02	2.11 ± 0.13	2.7 ± 0.2	0.97 ± 0.03	1.93 ± 0.13	2.96 ± 0.17
N_ε_(His237)–H_w_(Water)	4.65 ± 0.15	1.03 ± 0.03	1.96 ± 0.10	4.57 ± 0.14	1.04 ± 0.03	1.85 ± 0.09
O_γ_(Ser160)–H_w_(Water)	4.01 ± 0.11	2.32 ± 0.06	0.99 ± 0.03	4.06 ± 0.18	2.06 ± 0.11	0.99 ± 0.03
O_δ1_(Asp206)–H_δ_(His237)	3.6 ± 1.1	2.0 ± 0.3	2.4 ± 0.5	2.4 ± 0.5	1.9 ± 0.2	2.3 ± 0.4
O_δ2_(Asp206)–H_δ_(His237)	3.5 ± 1.1	2.7 ± 0.4	3.1 ± 0.5	3.3 ± 0.5	2.9 ± 0.5	3.4 ± 0.4
H(Met161)–O_2_(PET)	2.2 ± 0.3	1.9 ± 0.13	1.91 ± 0.13	1.97 ± 0.17	1.88 ± 0.15	2.05 ± 0.18
H(Tyr87) −O_2_(PET)	2.2 ± 0.3	1.9 ± 0.2	1.93 ± 0.15	1.95 ± 0.14	1.81 ± 0.11	1.88 ± 0.11

Prior to the detailed analysis of the PET degradation by FAST-PETase, it is convenient to briefly describe the reaction mechanism obtained for the wild-type PETase and compare our findings with those reported in the literature to validate our computational approach. The FEP calculated for wild-type PETase clearly indicates that acylation occurs through a single and concerted step involving a single transition state (**TS1**) (Figure 4). The free energy barrier was estimated to be 16.5 ± 0.2 kcal mol^–1^ in good accord with that experimentally determined for BHET (18.0–18.6 kcal·mol^–1^) and with those theoretically predicted for PET-like dimer models at different quantum-chemical levels (17.0 and 20 kcal·mol^–1^, respectively).^26,27^ For the deacylation step (Figure 5), a free energy barrier of 10.0 ± 0.2 kcal·mol^–1^ was predicted, which again is in line with the values calculated by similar methods (14.0 and 15.1 kcal·mol^–1^).^26,27^ These outcomes suggest that the selected computational approach is reliable for further studies of PETase-like variants.

Turning our attention to FAST-PETase, the predicted FEP for the acylation stage is significantly different compared to that obtained for PETase (Figure 4) with an important reduction of the energy barrier (12.1 ± 0.1 kcal mol^–1^). Starting from **RS**, the structure for the FAST-PETase:PET complex shows optimal contacts for the initiation of the chemical reaction; i.e., the distance between oxygen O_γ_ (Ser160) and carbon C_2_ (PET) is calculated to be relatively close (2.62 ± 0.06 Å) for a further nucleophilic attack and the Ser160 H_γ_ and His237 N_ε_ are also well oriented (distance of 1.87 ± 0.09 Å) for a proton transfer. H-bonds between oxygen O_2_ (PET) and hydrogen atoms of Tyr87 and Met161 are already set (Table 1 and Figure 4b). These distances are predicted to be similar for the wild-type PETase enzyme (Table 1 and Figure S9). The main difference between the enzyme:PET structures for the mutant and wild-type enzymes in **RS** lies in the O_δ1_–H_δ_ and O_δ2_–H_δ_ distances (Asp206 and His237), for which the fluctuation is higher in FAST-PETase compared to PETase in line with the previous analysis of the mobility of Asp206 (Figure 3).

From **RS** to the acyl-enzyme intermediate (**I1**), the reaction occurs through a broad transition state (**TS1**), which is significantly stabilized compared to the wild-type PETase (Figure 4). The **TS1** region comprises a wide variety of energetically-similar structures and begins when the H_γ_ is equally shared by Ser160 and His237 (ca. 1.2 Å) and the nucleophilic attack of oxygen O_γ_ (Ser160) to C_2_ (PET) is initiated (average C_2_–O_γ_ distance of 2.01 Å). From this point, the Ser160 gradually transfers its proton to the His237 with a clear H_γ_–O_γ_ (H_γ_–N_ε_) distance increase (decrease), whereas the C_2_–O_γ_ distance undergoes a steady shortening until it reaches a covalent bond of 1.35 ± 0.03 Å. On the other hand, the ester C_2_–O_1_ bond of the PET model exhibits a continuous lengthening from 1.35 ± 0.03 to 4.21 ± 0.08 Å. This C_2_–O_1_ lengthening is accompanied by a progressive shortening of the distance of the H_γ_ proton (now bound to His237) to the PET oxygen O_1_ due to a favorable electrostatic interaction between both atoms as a consequence of the C_2_–O_1_ bond cleavage. In all this **TS1** region, a tetrahedral structure for the PET C_2_ is predicted. Unlike the extended **TS1** region found for FAST-PETase, the wild-type PETase exhibits a well-defined tetrahedral **TS1** with C_2_–O_γ_ and C_2_–O_1_ distances of 1.40 ± 0.03 and 1.96 ± 0.06 Å, respectively. In addition, H_γ_ is bound to His237 and oxygen O_1_ of the PET model with similar distances (1.21 ± 0.11 and 1.34 ± 0.14 Å for H_γ_–N_ε_ and H_γ_–O_1_ distances, respectively).

For both FAST-PETase and PETase enzymes, the **TS1** state seems to be stabilized, in line with other experimental and computational studies,^24,27^ by the formation of an oxyanion hole between the amine groups of Met161 and Tyr87 and the oxygen O_2_ of the PET with short H–O_2_ contacts in the 1.8–1.9 Å range (Table 1, Figures 4b, and S9). The additional stabilization of **TS1** for the FAST-PETase mutant compared to the wild-type enzyme comes from the higher basicity of Asp206 within the FAST-PETase:PET complex as a consequence of the N233K mutation as previously discussed (Figure 3). This higher basicity (lower stabilization of the negative charge due to the lack of vicinal H-bonds) promotes a stronger electrostatic interaction with the protonated His237 in FAST-PETase (−47.0 ± 4.0 kcal mol^–1^) compared to PETase (−44.0 ± 3.0 kcal mol^–1^). A structural signature of this optimal electrostatic interaction for FAST-PETase is the bifurcated H-bond interaction established between the two Asp206 oxygen atoms (Ο_δ1_ and O_δ2_) and the His237 H_δ_ along the **TS1** region with average distances in the 2.0–2.5 Å range in both cases (Table 1 and Figure S10). In contrast, only one H-bond (O_δ1_–H_δ_) can be set for PETase during the acylation (Table 1 and Figure S10). Therefore, the higher basicity of Asp206 and, consequently, the more favorable His237-Asp206 electrostatic interaction predicted for FAST-PETase is the origin of the free energy barrier decrease.

Finally, **TS1** evolves to the **I1** intermediate through the breaking of the ester C_2_–O_1_ bond (4.21 ± 0.08 Å for **I1**) and the H_γ_–O_1_ bond formation (0.97 ± 0.02 Å) for FAST-PETase. In **I1**, the acyl-enzyme complex is also stabilized due to the H-bonds between oxygen O_2_ of PET and the amine groups of Tyr87 and Met161 (contacts in the 2.0–2.1 Å range, Table 1). A similar **I1** structure is found for PETase (Table 1 and Figure S9). The difference arises from the fact that the **I1** intermediate is predicted to be slightly exergonic for FAST-PETase and endergonic for PETase.

For the deacylation stage (Scheme 1), a water molecule, introduced in the active site and well oriented with respect to the catalytic triad and the acyl-enzyme complex, can initiate the reaction (starting from **I2**) by a simultaneous proton transfer to His237 and a nucleophilic attack to the PET carbonyl (C_2_) of the enzyme:substrate adduct. This would generate a new tetrahedral transition state (**TS2**), which would evolve toward the product (**PS**) by means of a proton transfer from His237 to Ser160 (regenerating the enzyme scaffold) and the release of the TPA-terminal PET fragment (Scheme 1). Note that, as the active site of the PETase-like variants is located on the enzyme surface, water molecules are easily accessible for the deacylation step, in particular when one of the substrate fragments has left the active site.

For FAST-PETase, the active water in the **I2** intermediate is found to be relatively far from the acyl-enzyme adduct (Table 1 and Figure 5) with distances between the water oxygen (O_w_) and carbon C_2_ (PET) of 3.68 ± 0.05 Å. The water H_w_ and the His237 N_ε_ are computed to be at 4.65 ± 0.15 Å, which is also rather remote for a direct proton transfer. On the other hand, the **I2** intermediate does not preserve short contacts between Asp206 and His237 (O_δ1_–H_δ_ and Ο_δ2_–H_δ_ distances of 3.6 ± 1.1 and 3.5 ± 1.1 Å, respectively). **I2** also exhibits a favorable H-bonding interaction between oxygen O_2_ (PET) and the Tyr87 and Met161 hydrogen atoms with similar distances (2.2 ± 0.3 Å for both). For the wild-type PETase, the structure of **I2** is similar to that obtained for FAST-PETase (Table 1 and Figure S9).

As the reaction advances from **I2**, we can differentiate three relevant regions (Figure 5a): an initial stable intermediate, the transition state (**TS2**), and the products (**PS**) together with the enzyme recovering. The deacylation reaction presents a free energy barrier for FAST-PETase (PETase) of 11.2 ± 0.1 kcal mol^–1^ (10.0 ± 0.2 kcal mol^–1^) and an exergonic free energy of −6.7 ± 0.1 kcal mol^–1^ (−4.4 ± 0.2 kcal mol^–1^). Unlike the acylation stage, the deacylation shows clear minima for both enzymes, which can be ascribed to the favorable interaction between the TPA-terminal PET fragment and the enzyme in the active sites. As can be inferred from Figure 5a, the initial intermediate for FAST-PETase is steadily destabilized (∼2.5 kcal mol^–1^), whereas there is almost no energy penalty to reach this intermediate (a tiny energy barrier of ∼0.9 kcal mol^–1^) for PETase. For both enzymes, this initial and stable intermediate results from an optimal orientation of the active water molecule for the nucleophilic attack and proton transfer; in particular, a disposition with a significant shorter distance compared to **I2** between the active water and His237 (N_ε_–H_w_ of ca. 2.0 Å). The energetic destabilization of this intermediate for FAST-PETase is in line with the higher electrostatic interaction felt by the reactive water molecule as it approaches the PET fragment compared to PETase (Figure S11). These findings reveal that the free energy barrier for the deacylation step in FAST-PETase can be decomposed in two processes with different energy costs: i) the insertion and orientation of the reactive water molecule (∼2.5 kcal mol^–1^) and ii) the formation of the tetrahedral transition state (∼8.7 kcal mol^–1^). For PETase, the free energy barrier comes mainly from the tetrahedral transition state formation (∼10.0 kcal mol^–1^). Therefore, there is even a slight energy stabilization of the transition state for FAST-PETase during the deacylation stage, which, in line with acylation, also comes from the favorable electrostatic interaction between the protonated His237 and Asp206.

From the **I2** intermediate, the reaction progresses until **TS2**. Note that, for convenience and comparison purposes, we have kept the label TS2 in Table 1 and Figure 5 for a structure that, in the case of FAST-PETase, is not a proper transition state but a stable high-energy intermediate. For FAST-PETase, **TS2** exhibits a clear tetrahedral structure similar to **TS1**. The C_2_–O_γ_ distance (C_2_ of PET and O_γ_ of Ser160) has undergone a slight lengthening (1.49 ± 0.05 Å), whereas a short contact between the PET fragment and water hydroxyl has appeared (C_2_–O_W_ of 1.49 ± 0.05 Å). At **TS2**, the proton transfer from the active water to His237 has totally occurred with a N_ε_–H_w_ distance of 1.03 ± 0.03 Å and a significant lengthening of O_w_–H_w_ (2.11 ± 0.13 Å). Likewise, a reorientation of Ser160 and His237 for a subsequent proton transfer (now from His237 to Ser160) has taken place in **TS2** with an O_γ_–H_w_ contact of ca. 2.3 Å (Figure 5b). For the wild-type PETase, **TS2** shows a slightly weaker tetrahedral character with longer C_2_–O_W_ distances (1.80 ± 0.05 Å) compared to FAST-PETase (1.49 ± 0.05 Å). The proton transfer from the active water to His237 has also happened (1.04 ± 0.03 Å), although the lengthening of O_w_–H_w_ is smaller (1.93 ± 0.13 Å). Regarding the oxyanion hole H-bonds, and in line with what was found for **I2**, two significant H-bonds between the oxygen PET O_2_ and Tyr87 and Met161 H atoms are predicted and slightly strengthened for both FAST-PETase and PETase (in the 1.8–1.9 Å range). Our findings, in contrast to those reported by Jerves et al., therefore indicate that the nucleophilic water attack and deprotonation processes are asynchronous, being the His237 protonation the first important event for the deacylation stage for both enzymes.

In the products (**PS**), the bond between the PET fragment and Ser160 (C_2_–O_γ_) has significantly lengthened (2.78 ± 0.05 Å) for FAST-PETase, and the Ser160 has been regenerated due to the proton transfer from His237 to Ser160 (O_γ_–H_w_ and N_ε_–H_w_ distances of 0.99 ± 0.03 and 1.96 ± 0.10 Å, respectively). Additionally, the relevant C_2_–O_w_ covalent bond has been formed (1.33 ± 0.03 Å), generating the TPA-terminal PET fragment. Compared to FAST-PETase, a comparable **PS** structure for PETase is calculated with similar distances (Table 1 and Figure S9).

## Conclusions

In this article, we perform a detailed computational study to gain insights into the enhancement of the PET degradation biocatalyzed by the promising FAST-PETase enzyme. In particular, we seek to disentangle the role that the five mutations incorporated in the FAST-PETase scaffold (S121E/D186H/R224Q/N233K/R280A), all of them far from the active site, play on the stabilization of the initial PET-enzyme complex or other important intermediates (transition states) determining the catalytic reaction rate for the PET biodegradation. With this aim, we have applied classical and hybrid (QM/MM) MD simulations for the wild-type PETase and mutant FAST-PETase as the enzyme scaffolds and a PET model (trimer) as the substrate to understand the catalytic mechanism. The free energy landscapes for the reaction mechanism have been predicted by using the ASM together with umbrella-sampling simulations.

Our outcomes support that the FAST-PETase variant follows the canonical reaction mechanism of the wild-type PETase and other hydrolases with two stages (acylation and deacylation), in which a Ser-His-Asp triad is actively involved in the biocatalytic process. FAST-PETase displays a catalytic mechanism in which both acylation and deacylation exhibit broad transition state-like regions, the acylation stage being the rate-limiting reaction step with a free energy barrier of 12.1 kcal mol^–1^. That free energy barrier is significantly smaller than that predicted for PETase (16.5 kcal mol^–1^), confirming that FAST-PETase is a better biocatalyst for PET degradation. Our study reveals that the origin of the enhanced activity of FAST-PETase mainly comes from the N233K mutation, and is not due to the formation of a salt bridge interaction, as previously suggested,^16^ but rather to an opposite effect. In particular, the lack of a salt bridge interaction between Lys233 and Glu204 gives rise to a change of the chain folding of the residues around Glu204, among which is Asp206 of the catalytic triad. This folding hinders that Asp206 establishes effective H-bonds with its vicinal residues (Ile208 and Ala209) compared to PETase and, as a consequence, Asp206 acquires a more basic character favoring the interaction with the protonated His237 in the transition state of the acylation stage. This improved His237-Asp206 interaction lowers the free energy barrier of acylation and, consequently, accelerates the PET depolymerization.

The results here presented therefore rationalize the molecular origin of the improvement of the catalytic activity of FAST-PETase in PET degradation. Additionally, we believe that the enhanced basicity of Asp206, indirectly caused by the N233K mutation, might also be the reason why other PET-degrading enzymes incorporating the N233K mutation show an increased catalytic activity. Overall, our study contributes to an in-depth comprehension of the PET biodegradation process and can help for the rational design of novel PET-hydrolyzing enzymes for feasible industrial applications.

## Supporting Information Available

Evolution of the selected distances between Lys233 and Glu204; representation of the Asp206 loop change; evolution of the H-bond distances between Asp206 and Ile208 and Ala209; probability distribution function of the electrostatic potential felt by Asp206 oxygen atoms; representation of the cleft distance measurement; radial distribution function of water molecules around Ser160 and number of water molecules within a cavity created from the catalytic triad for both enzyme:PET complexes; hydrophobic surface areas for both enzyme:PET complexes; representative structures of the active site at the relevant points for PETase; O_δ1_(Asp206)–H_δ_(His237) and O_δ2_(Asp206)–H_δ_(His237) distances along the MFEP; evolution of the electrostatic interaction (Δ*E*_Elec_) of the reactive water during the first stage of deacylation (PDF)
