# Supplementary material for: Insights into the Enhancement of the Poly(ethylene terephthalate) Degradation by FAST-PETase from Computational Modeling
Source: J Am Chem Soc. 2023 Aug 16;145(35):19243–55. doi: 10.1021/jacs.3c04427 (PMC10851425; doi:10.1021/jacs.3c04427)
Supplement: Supplementary file 1 — ja3c04427_si_001.pdf [file ja3c04427_si_001.pdf]

Supporting Information

Insights into the Enhancement of the Poly(ethylene terephthalate) (PET) Degradation by FAST-PETase from Computational Modeling

Rafael García-Meseguer,† Enrique Ortí,† Iñaki Tuñón,‡ J. Javier Ruiz-Pernía,‡ and Juan Aragó*,†

† Instituto de Ciencia Molecular (ICMol), Universidad de Valencia, Catedrático José Beltrán 2, Paterna, 46980, Spain.

‡ Departamento de Química Física, Universidad de Valencia, 46100 Burjassot, Spain.

* Corresponding Author E-mail: juan.arago@uv.es

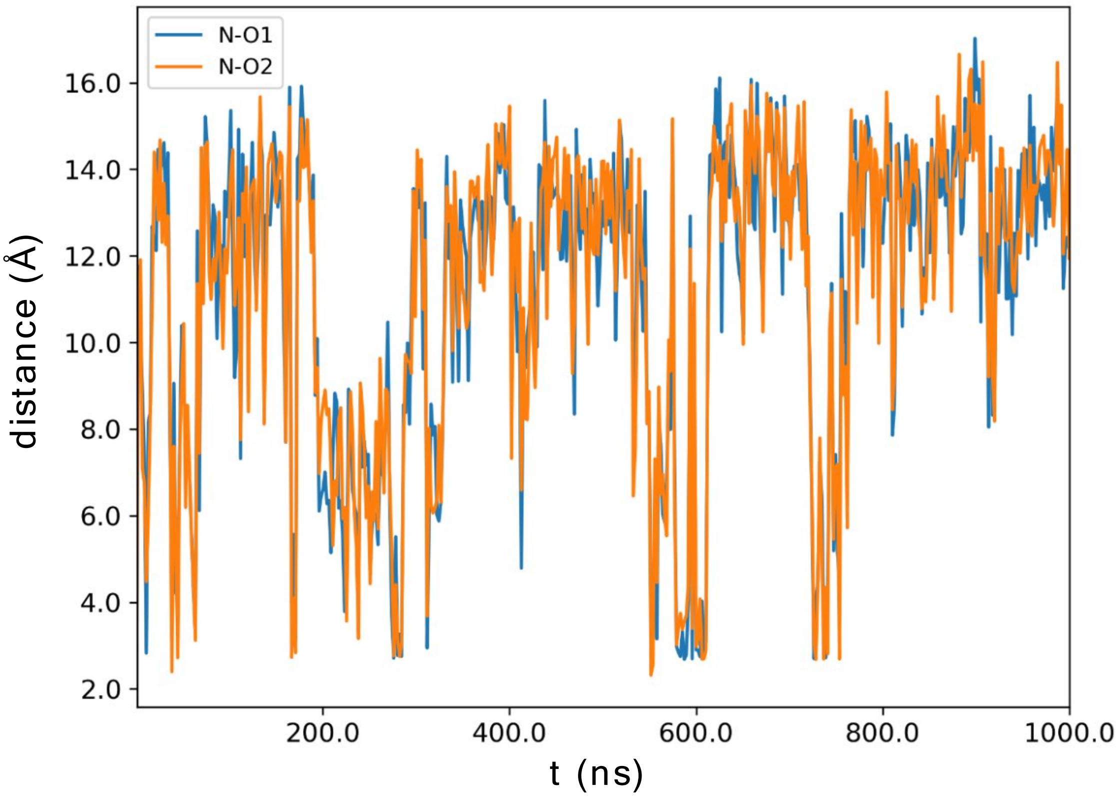

Figure S1. Evolution of the distance along the MD simulation between the positively-charged nitrogen of Lys233 with the two oxygen atoms of Glu204.

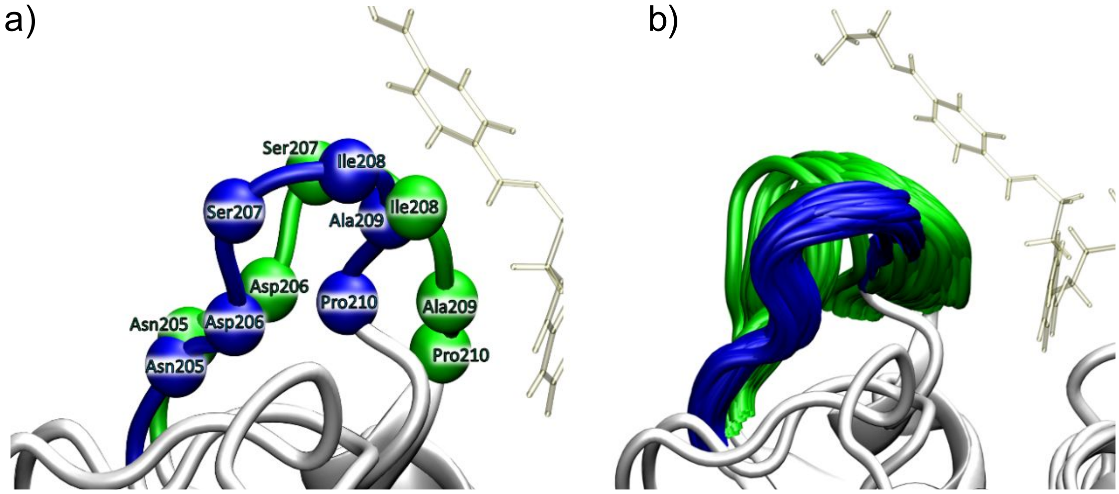

Figure S2. a) Comparison of the Asp206 loop (residues 205-210) for FAST-PETase:PET (green) and PETase:PET (blue) complexes. Each residue of the loop is depicted and labeled by the location of its C_{α} as balls. b) Representation of multiple structures of the loop taken from the MD enzyme:PET trajectories.

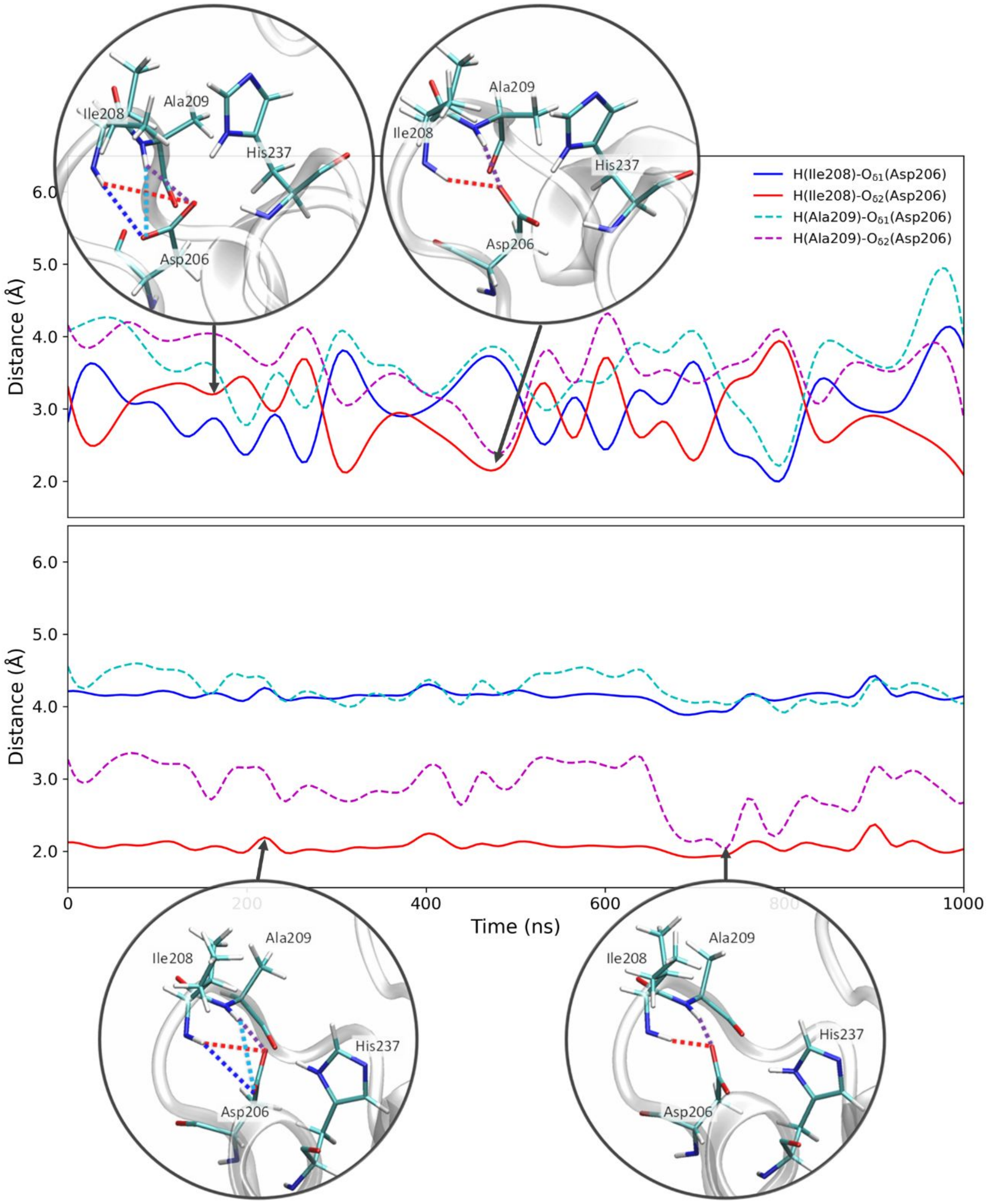

Figure S3. Plot of the average distances (each 500 ps) between Ile208 H atom and Asp206 $O_{\delta1}$ or Asp206 $O_{\delta2}$ atoms during the classical trajectories for FAST-PETase:PET (top) and PETase:PET (bottom) complexes. Snapshots of the trajectories show the relevant residues labeled.

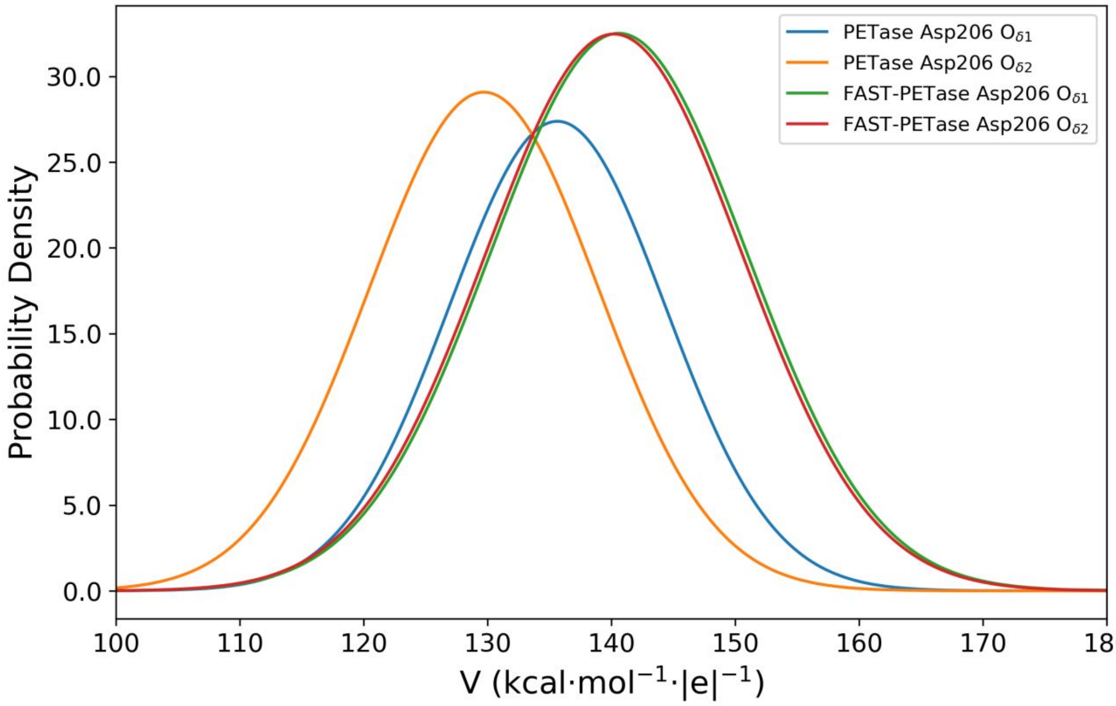

Figure S4. Probability distribution function of the electrostatic potential (V) felt by the oxygen atoms of Asp206 ($O_{\delta 1}$ and $O_{\delta 2}$) during the classical trajectories for the FAST-PETase:PET and PETase:PET complexes for the reactant state (**RS**) in the acylation step.

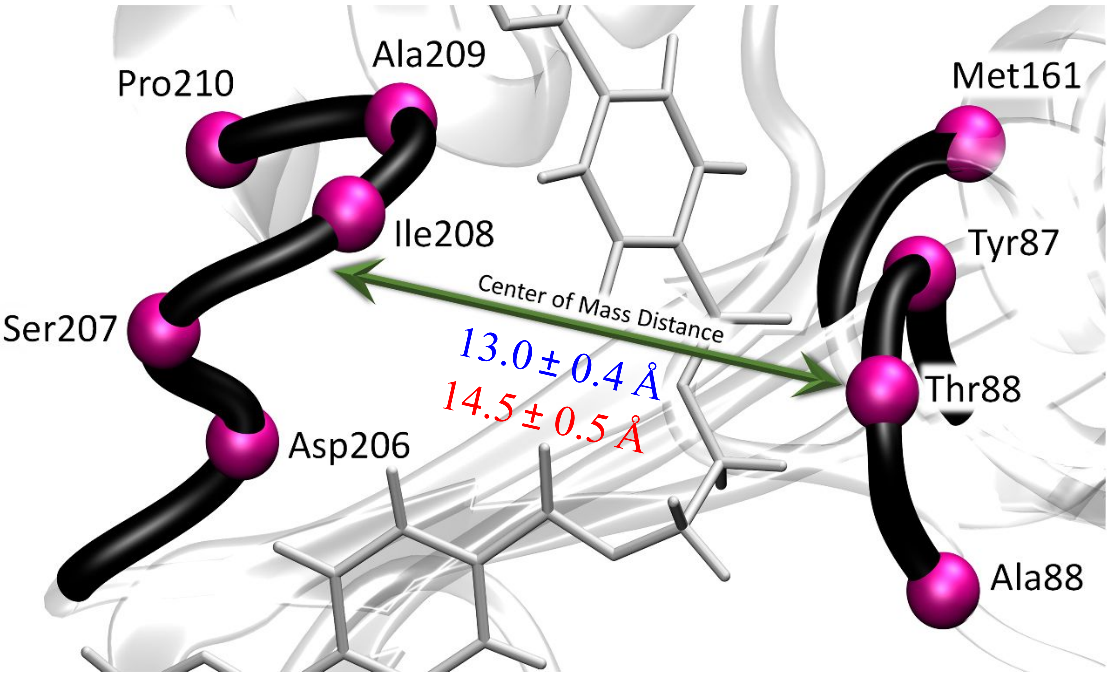

Figure S5. Estimation of the cleft distance using the center of mass of the C_α (drawn as magenta balls) of the labeled residues for FAST-PETase (blue) and PETase (red).

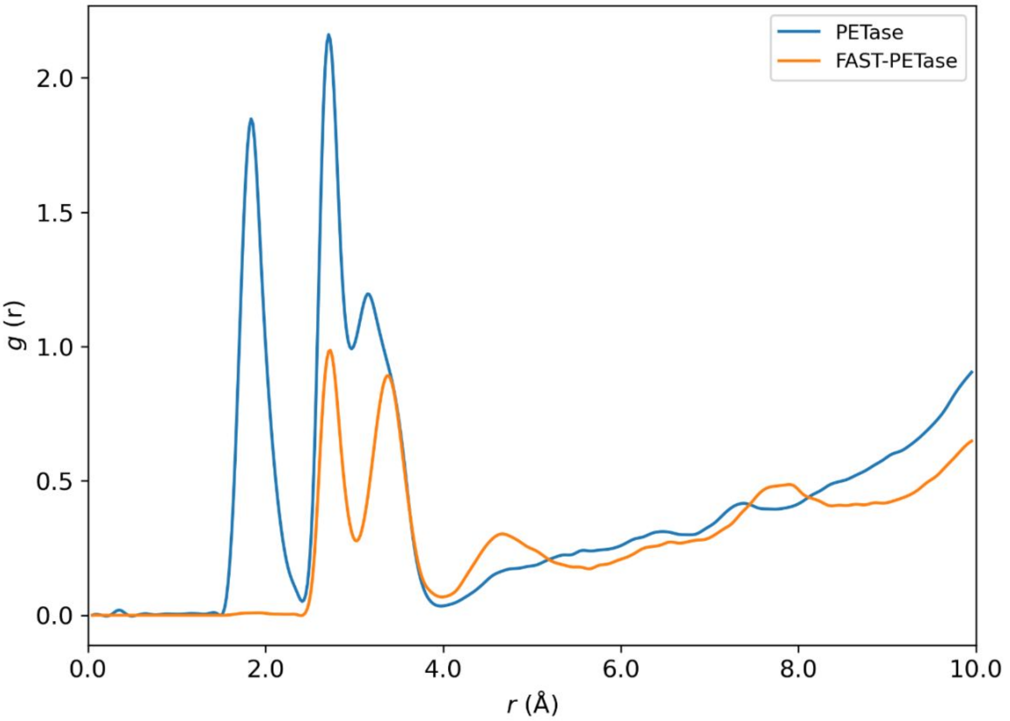

Figure S6. Radial distribution function for the water molecules starting from Ser160 O_γ atom estimated for the FAST-PETase:PET and PETase:PET complexes computed along the classical MD simulation.

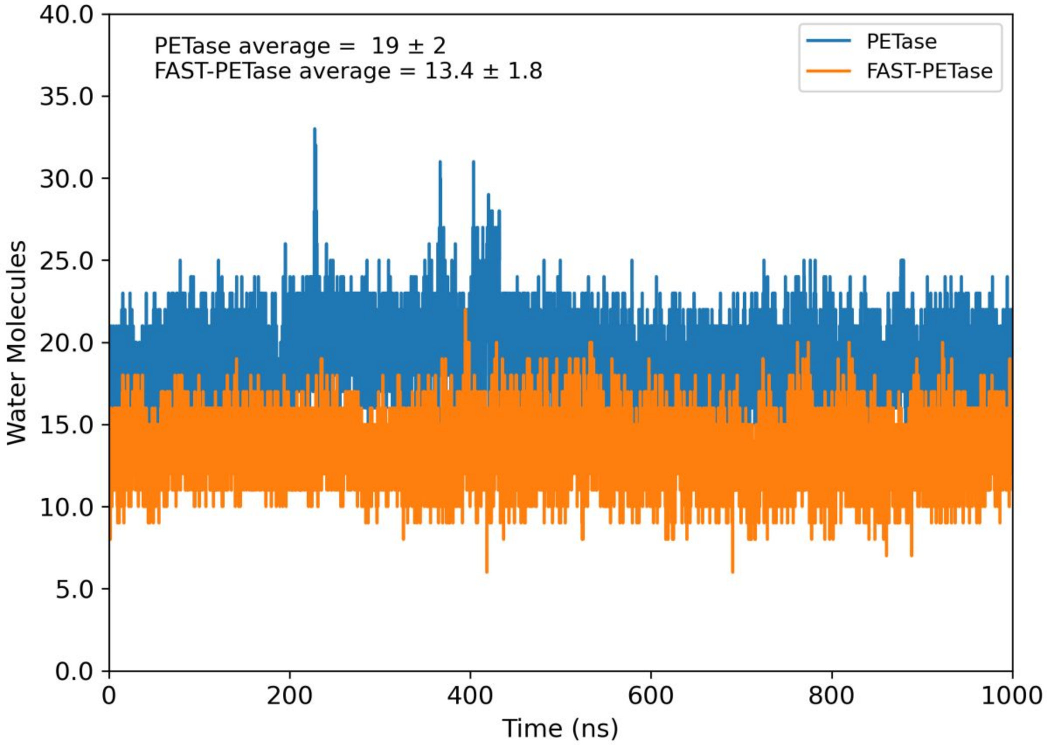

Figure S7. Time evolution of the number of water molecules within a cavity created from the catalytic triad (Ser160-His237-Asp206) with a radius of 3.4 Å from each residue computed for the FAST-PETase:PET and PETase:PET complexes along the classical MD simulation.

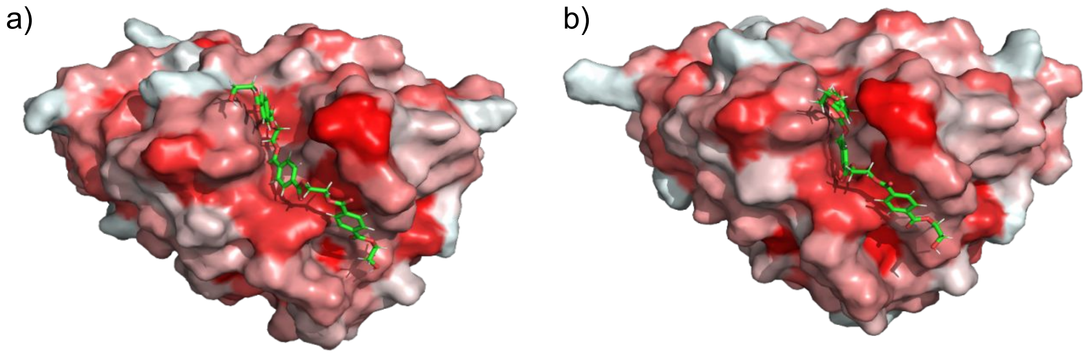

Figure S8. Image of the accessible surface area of FAST-PETase (a) and PETase (b) with the PET substrate included (sticks drawing) as a reference for a snapshot of a reactant enzyme:PET complex. The coloring of the surface represents the hydrophobicity of the residues, the red color highlighting the more hydrophobic residues.

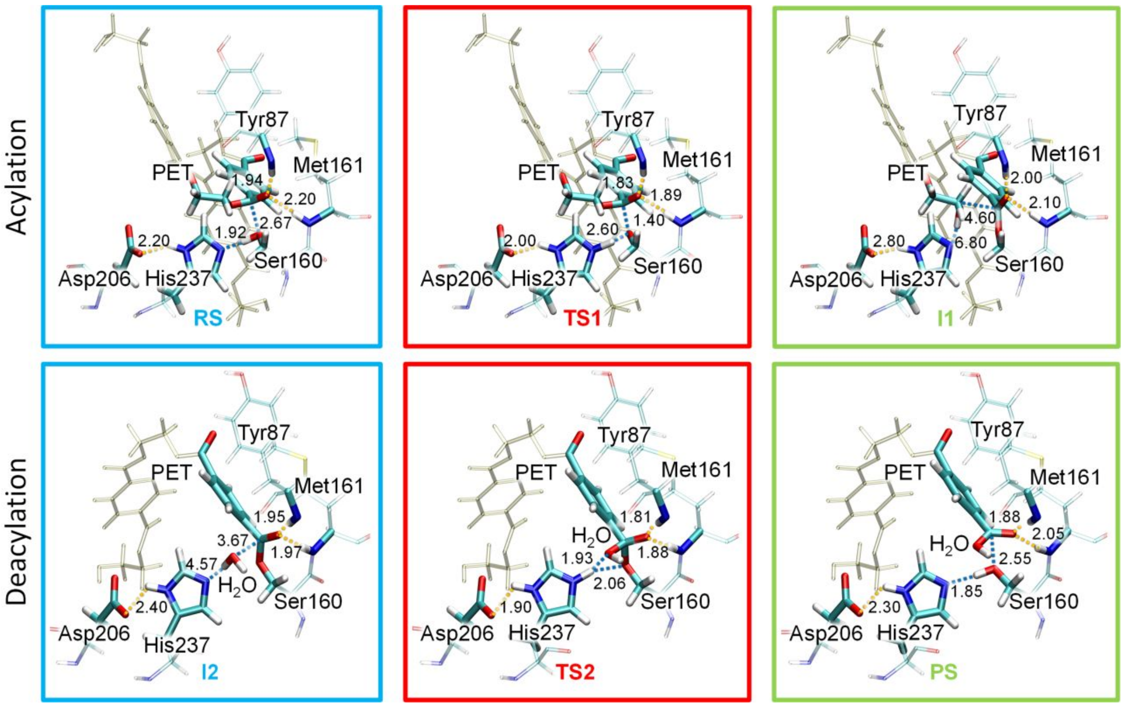

Figure S9. Representative structures (atom distances in Å) of the active site at the relevant points for the acylation (**RS**, **TS1**, and **I1**) and deacylation (**I2**, **TS2**, and **PS**) stages predicted for PETase.

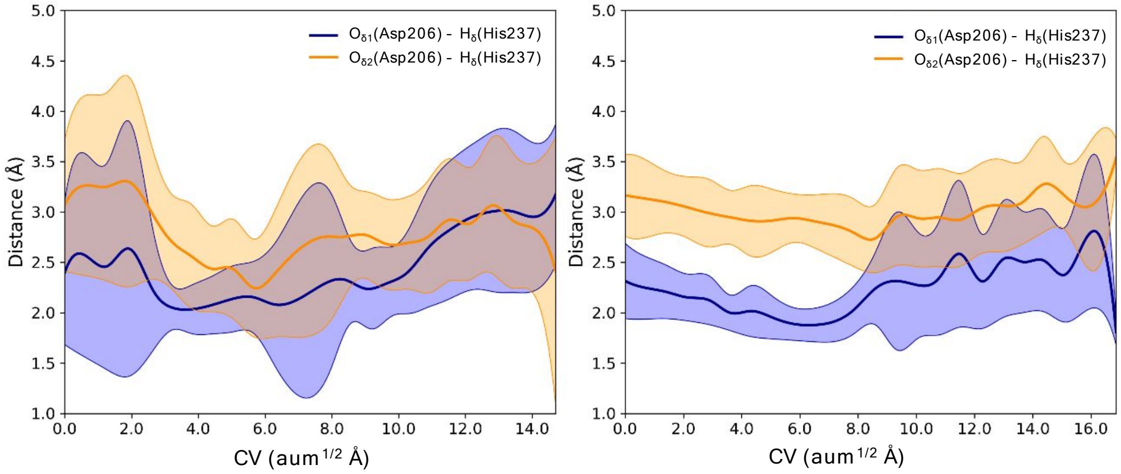

Figure S10. Evolution of the $O_{\delta1}$(Asp206) − H_δ(His237) and $O_{\delta2}$(Asp206) − H_δ(His237) distances along the acylation stage for FAST-PETase (left side) and PETase (right side). The shaded area represents the standard deviation.

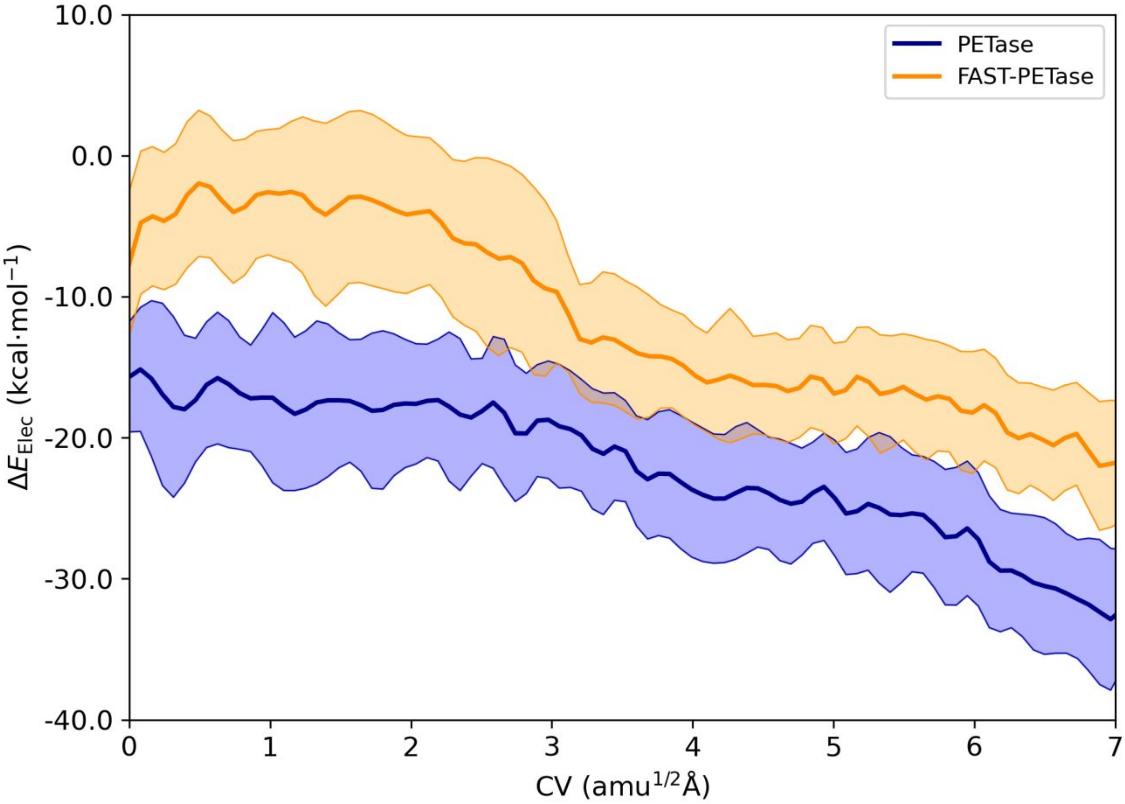

Figure S11. Evolution of the electrostatic interaction (ΔE_{Elec}) of the reactive water during the approximation to the PET fragment in the deacylation stage estimated for the FAST-PETase:PET and PETase:PET complexes. The shaded area represents the standard deviation.
